# Serum vascular endothelial growth factor has diagnostic and prognostic significance in ulcerative colitis

**DOI:** 10.55730/1300-0144.5841

**Published:** 2024-05-17

**Authors:** Musa SALMANOĞLU, İrfan KÜÇÜK, Başak ÇAKIR GÜNEY, Betül DOĞANTEKİN, Nurgül TÜKEL, Zeliha SERİNDAĞ, Habip YILMAZ, Mustafa KAPLAN

**Affiliations:** 1 Department of Internal Medicine, Sultan 2. Abdülhamid Han Training and Research Hospital, Health Sciences University, İstanbul, Turkiye; 2 Department of Gastroenterology, Kartal Dr. Lütfi Kırdar Training and Research Hospital, Health Sciences University, İstanbul, Turkiye; 3 İstanbul Provincial Health Directorate Public Hospitals Services Presidency, İstanbul, Turkiye; 4 Department of Gastroenterology, Sultan 2. Abdülhamid Han Training and Research Hospital, Health Sciences University, İstanbul, Turkiye

**Keywords:** Ulcerative colitis, vascular endothelial growth factor (VEGF), calprotectin

## Abstract

**Background/aim:**

In ulcerative colitis (UC), serum vascular endothelial growth factor (sVEGF) concentrations are elevated and there are conflicting results about serum calprotectin (SCP) and sVEGF as biomarkers. We aimed to evaluate the relationship between sVEGF and SCP levels in UC patients and the associations of these molecules with the phenotypes of UC.

**Materials and methods:**

This prospective case-control study included 60 UC patients and 30 healthy controls. The Mayo Clinical Score (MCS) was used to evaluate patients’ clinical features and the Mayo Endoscopic Score (MES) was used to evaluate endoscopic features of the cases. The method proposed by Truelove and Richards was applied in calculating the histology activity index (HAI). Human sVEGF (Cat.E0080Hu) and human calprotectin (Cat.E4010Hu) kits were used for the enzyme-linked immunosorbent assay (ELISA) measurements of sVEGF and SCP levels.

**Results:**

The median sVEGF and SCP levels were higher in the patient group compared to the healthy control group [2139 ng/L (126–5783) vs. 888 ng/L (715–5270), p = 0.002 and 932 ng/L (99–2648) vs. 80 ng/L (56–920), p < 0.001, respectively]. There was a strong correlation between SCP and sVEGF values (rho = 0.819, p < 0.001). The MCS, MES, and HAI values were positively correlated with sVEGF and SCP concentrations.

**Conclusion:**

sVEGF and SCP may be valuable auxiliary biomarkers for UC.

## 1. Introduction

The pathogenesis of ulcerative colitis (UC) remains elusive and is multifactorial. The cornerstone of UC pathogenesis is immune dysregulation. However, there is increasing interest in a variety of molecules related to pathogenicity that might account for the differences observed in the disease’s progression [1].

Angiogenesis is the growth of new blood vessels, mimicking an embryogenesis process [2]. Vascular endothelial growth factor (VEGF) is a member of the growth factor family, and it exerts angiogenic and antiapoptotic properties and takes part in pathological conditions [2–5].

Elevated serum levels and increased tissue expressions of VEGF in patients with inflammatory bowel diseases (IBDs) were reported in previous studies [6–9]. VEGF is also proposed as a therapeutic target in IBDs [5,10,11]. While the pathogenic functions of VEGF are well documented in experimental colitis, its relationships with the clinical, endoscopic, biochemical, and histological characteristics of UC have not been comprehensively established in clinical trials.

S100 family proteins lead to proinflammatory cytokine production that stimulates neutrophil, monocyte, and macrophage recruitment into inflammation sites, and they are released or actively secreted from cells and activate pattern recognition receptors (PRR). Thus, they can regulate innate and adaptive immune responses [12].

Calprotectin (CP; S100A8/A9) is a calcium-binding protein of the S100 family. It induces the nuclear factor κB (NF-κB) pathway [12,13]. Besides monocytes, dendritic cells, and vascular endothelial cells, CP is expressed abundantly in neutrophils [13,14]. Serum CP (SCP) was found to generate the inflammatory burden in patients with IBDs and to correlate with endoscopic activity in UC patients. However, conflicting results exist between SCP and IBDs [15–18].

Reliable markers that can be used as substitutes for colonoscopy and reflect the severity of mucosal inflammation are needed in UC. In this study, we evaluate the relationship between serum VEGF (sVEGF) and SCP levels in UC patients, as well as the associations between these molecules and disease phenotypes, including the severity of mucosal inflammation.

## 2. Materials and methods

### 2.1. Study population

The study included 60 patients with UC and 30 healthy controls admitted to our institute’s gastroenterology and internal disease department between June 2023 and December 2023. The local ethics committee approved the study (2023/86). Written informed consent was obtained from all participants.

Participants with clinical conditions that can affect sVEGF and SCP levels, such as sepsis, any malignancies, severe organ failure including chronic renal and liver diseases, acute or chronic infections, uncontrolled diabetes, autoimmune diseases, pregnancy and breastfeeding, nonsteroidal antiinflammatory drug use, or a history of intestinal resection were excluded from the study. The healthy control group included participants who underwent a colonoscopy for indications other than IBDs and whose colonoscopy results were normal.

Disease duration, medications for IBDs, comorbidities, family history of IBDs (IBDs in first-degree relatives), extraintestinal manifestations, and smoking habits were recorded for UC patients. Erythrocyte sedimentation rate (ESR), C-reactive protein (CRP), and other biochemical variables were measured before colonoscopy. After endoscopic examination, the Mayo Clinical Score (MCS) and Mayo Endoscopic Score (MES) were calculated.

### 2.2. Assessment of clinical and endoscopic variables in ulcerative colitis patients

For UC patients, the MCS was calculated. Possible scores range between 0 and 12. Scores of ≤2 were classified as indicating clinical remission, whereas scores of >2 indicated activation [19]. The disease extent of UC patients was defined in agreement with the Montreal classification. Proctitis and left-sided colitis were recorded as limited disease, whereas extensive disease and pancolitis were recorded as extensive disease. The MES was calculated to evaluate the endoscopic activation of UC and scores were determined as reflecting remission (0) or mild (1), moderate (2), or severe (3) colitis. Scores of 0 and 1 were recorded as inactive disease, whereas scores of 2 and 3 were recorded as active disease [19].

### 2.3. Histopathological evaluation of ulcerative colitis

Formalin-fixed, paraffin-embedded, and H&E-stained colonic biopsy samples of patients with UC were evaluated by the same pathologist who was blinded to the participants. The pathologist performed grading using a scale similar to that developed by Truelove and Richards [20]. The components of the scale were active inflammation (0–3 points), chronic inflammation (0–2 points), and crypt distortion (0–3 points). The histology activity index (HAI) score was taken as the sum of the scores of these components. The total possible HAI score was eight, and scores of <5 were recorded as reflecting histological remission, whereas scores of ≥5 reflected activation [20].

### 2.4. Measurement of sVEGF and SCP concentrations

After colonoscopy, serum samples for sVEGF and SCP measurements were separated from venous blood samples. After centrifugation at 5000 × *g* for 10 min at 30 °C, supernatant serum samples were stored in Eppendorf tubes at –80 °C for five months until analysis. For the enzyme-linked immunosorbent assay (ELISA) measurement of sVEGF, the commercially available Bioassay Technology Laboratory Human Vascular sVEGF ELISA Kit (Cat. No. E0080Hu, Lot: 202303011; BT Lab, Shanghai, China) was used (intraassay coefficient of variation: <8%, interassay coefficient of variation: <10%) with a microplate reader (BioTek Epoch 2 Microplate ELISA Reader, Agilent Technologies, Santa Clara, CA, USA). The commercially available Human Calprotectin Bioassay Technology Laboratory Kit (Cat. No. E4010Hu, Lot: 202303012; BT Lab) was used for the ELISA measurement of SCP (intraassay coefficient of variation: <8%, interassay coefficient of variation: <10%) with a microplate reader (BioTek Epoch 2 Microplate ELISA Reader, Agilent Technologies).

### 2.5. Statistical analysis

Data analysis was performed with IBM SPSS Statistics 26.0 (IBM Corp., Armonk, NY, USA). For descriptive data, numbers and percentages were used for categorical variables and mean ± standard deviation or median with range were used for continuous variables. Variables were investigated using the Kolmogorov–Smirnov or Shapiro–Wilk test to determine whether they were normally distributed. The chi-square test was used to compare categorical variables in different groups. The Student t-test or Mann–Whitney U test was used to compare continuous variables between two groups as appropriate.

While investigating the associations between continuous variables, correlation coefficients and their significance were calculated using the Pearson or Spearman test. The capacity of sVEGF and SCP values in predicting the presence of UC was analyzed using receiver operating characteristic (ROC) curve analysis. When a significant cutoff value was observed, the sensitivity, specificity, and area under the curve (AUC) values with the 95% confidence interval (CI) were presented. Values of p < 0.05 were considered to reflect statistically significant results (p < 0.05).

## 3. Results

In total, 60 UC patients (43 men and 17 women) and 30 healthy controls (22 men and 18 women) participated in the study. The groups were similar with respect to age and sex. Men dominated in both groups. CRP, ESR, and platelet values were higher among UC patients, whereas leukocyte and neutrophil values were similar between the groups. It was determined by colonoscopy that 68.3% of the patients with UC had limited disease and 55% had active disease. According to the MCS, most UC patients were in the clinically active phase, and 58.3% were under treatment. The median HAI score was 6 among the patients with UC. The participants’ demographic, clinical, and laboratory characteristics are presented in Table 1.

The median sVEGF and SCP concentrations were significantly higher in the UC group compared to the healthy controls [2139 ng/L (126–5783) vs. 888 ng/L (715–5270), p = 0.002 and 932 ng/L (99–2648) vs. 80 ng/L (56–920), p < 0.001, respectively] (Table 1). The patients who were in the clinically active phase had higher sVEGF and SCP values compared to those in remission [2680 ng/L (273–5783) vs. 793 ng/L (126–5368), p < 0.001 and 1100 ng/L (100–2524) vs. 340 ng/L (101–2648), p = 0.001, respectively]. Patients with UC having extensive disease also had higher sVEGF and SCP values compared to the patients with limited disease [2875 ng/L (840–5783) vs. 1867 ng/L (126–5368), p = 0.001 and 1313 ng/L (150–2524) vs. 801 ng/L (99–2648), p = 0.012, respectively] (Table 2). With respect to sVEGF and SCP concentrations, there was no statistically significant difference between the patients with and without a family history of IBDs and extraintestinal manifestations, and the concentrations were also not different between UC patients under treatment and those not under treatment (Table 2).

There was a strong correlation between SCP and sVEGF values (rho = 0.819, p < 0.001). The MCS, MES, and HAI values positively correlated with SCP and sVEGF values (Table 3). Platelet, CRP, and ESR values also positively correlated with SCP and sVEGF, whereas there was no correlation between leukocyte and neutrophil counts and the concentrations of SCP and sVEGF (Table 3).

The ROC curve of SCP concentrations had diagnostic accuracy for UC with an optimum cutoff level of 436.8 ng/L (AUC = 0.921, p < 0.001, sensitivity of 78.3%, sensitivity of 90.0%) (Figure). The ROC curve of sVEGF concentrations had diagnostic accuracy for UC with an optimum cutoff level of 1111.9 (AUC = 0.705, p = 0.002, sensitivity of 80.0%, sensitivity of 70.0%) (Figure).

## 4. Discussion

The severity of disease presentation may be variable among patients with UC and the disease course might have differences between individuals [1]. The prevalence of IBDs is increasing worldwide, especially in developed countries. IBDs present geographic and ethnic variations.

Angiogenesis is a complex process and several cell types and cytokines mediate it. It occurs in both physiological and pathological conditions. VEGF and other angiogenesis-related molecules, including VEGF receptor, endoglin, and platelet endothelial cell adhesion molecule-1, were found to exacerbate enteric mucosal damage in chronic mucosal inflammation [2]. VEGF has been known as the main molecule of angiogenesis for decades, but the usefulness of sVEGF and the clinical traits of UC have yet to be elucidated [6–9].

As a result of ongoing inflammation, ulceration and granulation tissue accompanied by pathologic angiogenesis take place in the colonic mucosa of UC patients. Traditionally, histopathologic methods can be used to diagnose angiogenesis, but it has also been detected by narrow-band imaging colonoscopy [21].

In a previous report, mean sVEGF values were higher in UC patients than in patients with irritable bowel syndrome and healthy controls, and the UC clinical activity index (UCAI) and the Rachmilewitz endoscopic activity index were used for UC patients [7]. The mean sVEGF levels were higher in patients with active disease according to colonoscopy than those with inactive disease, and sVEGF levels positively correlated with the UCAI. In the same study, sVEGF expression levels were analyzed immunohistochemically using mucosal samples of UC patients. In patients with active disease, immunostaining was more evident than in those with inactive disease [7].

Algaba et al. [8] studied the concentrations of angiogenic factors including VEGF-A, VEGF-C, VEGF-D, and VEGF-receptors 1, 2, and 3 in serum and in the colonic mucosa culture supernatants of patients with active and quiescent IBDs. In that study, 37 UC patients and 28 healthy controls were evaluated, MCS and MES scores were calculated to measure clinical and endoscopic activity, as in our study, and the Riley index was used to measure the histological activity of UC patients [8,22]. In endoscopic evaluations, VEGF concentrations were higher in active UC patients than in patients with quiescent disease. Compared to healthy controls, VEGF-receptor 3 concentrations were significantly higher in patients with active UC, and VEGF-A and VEGF-C also correlated significantly with MCS. Serum levels of VEGF were higher in patients with higher histological activity scores. VEGF-A is known as the main regulator of pathologic angiogenesis. Higher sVEGF-A values can be attributed to pathological angiogenesis induced in patients with clinically active disease [2].

The results of this study highlight the predictive value of sVEGF and the relevant receptors for clinical, endoscopic, and histologic activities in patients with UC [8]. Instead of the Riley index, we applied the method of Truelove and Richards to measure histological activity in UC. Despite the different methods applied, sVEGF concentrations correlated with the severity of mucosal inflammation.

In this study, we have detailed the disease characteristics of UC patients. Consistent with the findings described above, MCS values were higher in patients with clinically active disease and extensive colitis by colonoscopy [7,8]. Furthermore, sVEGF concentrations positively correlated with MCS, MES, HAI, platelet, leukocyte, and neutrophil values. There were positive correlations between sVEGF and acute-phase reactants (APRs). Regarding the severity of inflammatory activity, increased concentrations of blood count parameters and APR values may point to higher levels of angiogenetic activity, leading to higher sVEGF values. Regarding family history for IBDs, treatment status, and extraintestinal manifestations, there was no statistically significant difference among the UC patients. However, cohorts with larger sample sizes might reveal significant results.

The blood supply provided by angiogenesis is mandatory for delivering nutrients and oxygen for mucosal healing and regeneration. Failure of inflammatory resolution in the mucosa leads to disorganized tissues that are vulnerable to ulceration and friability, along with induced angiogenesis. The cardinal symptom of UC is bloody diarrhea, and it can be partially due to the induced angiogenesis. Our results are parallel to those of previous reports. Several methods are used to assess the clinical, endoscopic, and histologic activity of UC cases. Although different methods were used in previous studies, sVEGF values are generally found to correlate with disease activity and they may be valuable, cheap, and easily applicable biomarkers for the monitoring of UC.

Recent reports have highlighted the therapeutic efficacy of inhibiting VEGF [5,10,11]. Thalidomide is an antiangiogenetic agent and it has been found to reduce sVEGF protein expression in a dose-dependent manner in the tissue samples of pediatric patients with Crohn disease [10]. VEGF is not only a mediator of angiogenesis but also a signaling mediator of inflammation [4]. Although the exact mechanisms are poorly understood, VEGF dysregulation is often associated with tissue injury and inflammation, as in UC [4,23]. In a recent report, PR1P, a 12-amino-acid VEGF-binding peptide, was suggested to be a possible therapeutic agent for UC. It was found to prevent the proteolytic degradation of VEGF and preserve VEGF signaling [4]. In light of the current data, it can be said that VEGF activation in IBD cases has many impacts with regard to treatment.

SCP is a proinflammatory molecule, and the direct measurement of neutrophil SCP in feces is a good choice for monitoring IBDs in clinical practice [14]. Although fecal calprotectin (FCP) is a valuable diagnostic and prognostic marker that can predict clinical relapse in IBD cases, its measurement is expensive and not easily applicable [24]. FCP can exhibit significant within-day and day-to-day variations and patients may sometimes be unwilling to provide fecal samples [16]. For these reasons, SCP may be an ideal calprotectin test. However, there are conflicting results about the usefulness of SCP in IBDs, and previous reports declared inconsistent correlations between FCP and SCP [13–18]. The diagnostic value of SCP and its correlations with the phenotypes of UC are yet to be elucidated.

Since SCP is a proinflammatory molecule, it is elevated in many chronic inflammatory diseases [25]. Although the exact origin of SCP is unknown, as a low-molecular-weight and small molecule, overexpression of calprotectin in the gut wall might diffuse into circulation in IBD cases [13]. In an experimental colitis model, SCP was closely correlated to macroscopic and microscopic disease scores [26].

In a study by Mori et al. [13], SCP values were evaluated in patients with UC (n = 97) and Crohn disease (n = 105) and healthy controls (n = 92). The MCS was calculated to measure the clinical activity of UC, but no correlation was found between SCP and MCS. On the other hand, SCP values were higher in CD patients with clinically active disease according to the Harvey–Bradshaw index. Most patients in our UC group were in the active phase, and this may partially explain the high SCP values in our study. Homogeneous cohorts with larger sample sizes might reveal more significant results regarding the MCS [13].

We noted higher SCP values in patients compared to healthy controls. SCP values were also higher in UC patients with clinically active disease than in those in remission, and the patients with extensive disease by colonoscopy had higher SCP concentrations than those with limited disease. In addition, we reported positive correlations between SCP and MCS, MES, and HAI.

Kalla et al. [15] reported positive correlations between CRP and SCP in IBD patients, and SCP was the strongest individual predictor of an IBD diagnosis (n = 96, both UC and CD). As in our study, higher SCP values were found in UC patients. In that study, a multiple-biomarker model was derived using multivariable logistic regression analysis and SCP was combined with other blood-based tests. SCP correlated strongly with the considered biomarkers, including FCP, and it was proposed as a novel diagnostic and prognostic marker for IBDs [15].

Carlsen et al. [16] examined SCP using cross-sectional and longitudinal data from adolescent UC patients. According to the cross-sectional data, SCP correlated with CRP, endoscopic, and symptom scores, and no significant correlation was reported between SCP and FCP. In contrast, there was no correlation among SCP, endoscopic, and symptom scores, but a significant correlation was noted between SCP and FCP based on the longitudinal data [16].

Yasuda et al. [27] examined SCP values in pediatric patients with UC (n = 77) and healthy controls (n = 22). The Pediatric Ulcerative Colitis Activity Index (PUCAI) was used to evaluate disease activity. Although SCP values were higher among active UC patients, there was no significant difference between the values of active and inactive UC patients and the healthy controls. To distinguish active disease from remission, AUC values for SCP were evaluated and found to be no greater than those for CRP or ESR. In our study, we also reported higher SCP values, and our results were statistically significant. The different results may be due to the ages of the patients and the different UC clinical activity indexes that were used in these studies.

Since UC has geographic and ethnic variations, the inconsistent results in the current literature might partially be due to those variations, and larger cohorts including participants from different regions of the world are needed to evaluate the usefulness of SCP in UC.

The severity of mucosal inflammation in UC is related to disease recurrence and long-term complications. Resolution of the inflammation in the colonic mucosa is the primary treatment goal of current medical treatments [28]. We noted positive correlations between sVEGF and SCP values and the severity of mucosal inflammatory activity in UC. In light of these results, sVEGF and SCP values might be ideal predictors for treatment response if they are evaluated prospectively.

As noted above, there is a link between VEGF and calprotectin activities [12,13]. Calprotectin induces interleukin-6 (IL-6) secretion via toll-like receptor four, and IL-6 leads to VEGF production [29]. Our study’s strong correlation between SCP and sVEGF values highlighted the association between sVEGF and calprotectin activity [29].

The major limitation of this study was the small size of the study population as it was a single-center trial. Larger cohorts might better show the relationship between characteristics of UC and SCP and sVEGF values. In addition, if we had measured FCP concentrations in UC patients, it might have provided valuable information about disease activity.

## 5. Conclusion

In UC, sVEGF may be a valuable auxiliary biomarker with respect to elevated sVEGF values. Studies from different ethnic groups and geographic regions might further delineate the diagnostic and prognostic accuracy of SCP. Diagnostic strategies with the possibility of therapeutic interventions can be developed by identifying new practical and objective biochemical markers in IBDs.

## Figures and Tables

**Figure:**
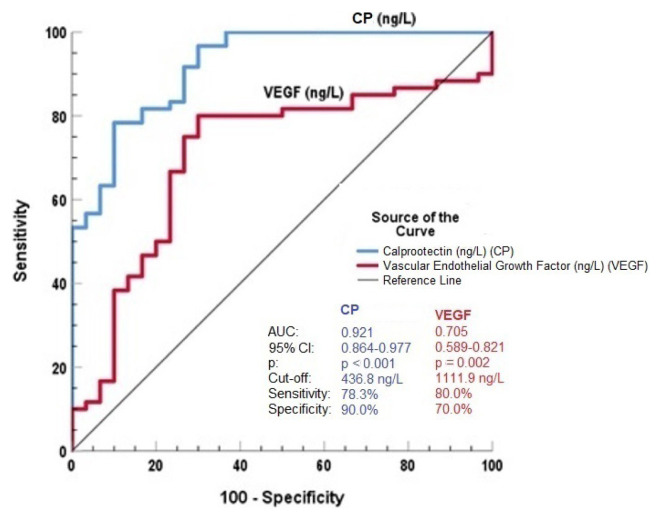
ROC curve analysis of the predictive values of SCP and sVEGF concentrations for ulcerative colitis.

**Table 1:** Demographic, clinical, and laboratory characteristics of the study population.

Demographic features	Ulcerative colitis	Control group	p
	n = 60	n = 30	
Sex, n (%)			
Female	17 (28.3)	8 (26.7)	0.868¥
Male	43 (71.3)	22 (73.3)	
Age (years), mean ± SD	43.3 ± 13.1	43.2 ± 10.6	0.966*
Disease duration (years), median (range)	2.25 (0–30)		
IBDs in first-degree relatives, n (%)	10 (16.7)		
Extraintestinal manifestations, n (%)	6 (10.0)		
ESR (mm/h), median (range)	30 (2–104)	14 (2–42)	<0.001&
CRP (mg/L), median (range)	11.4 (0.2–129.0)	3.0 (0.5–14.2)	<0.001&
Leukocytes (×10^3^/μL), median (range)	7.7 (4.3–19.0)	7.7 (3.2–14.5)	0.644&
Neutrophils (×10^3^/μL), median (range)	5.1 (2.3–13.0)	4.6 (1.6–12.2)	0.285&
Platelets (×10^3^/mm^3^) median (range)	309 (176–731)	241 (167–344)	<0.001&
Serum VEGF ( ng/L), median (range)	2139 (126–5783)	888 (715–5270)	0.002&
Serum calprotectin (ng/L), median (range)	932 (99–2648)	80 (56–920)	<0.001&
Extent of ulcerative colitis, n (%)			
Limited disease	41 (68.3)		
Extensive colitis	19 (31.7)		
Mayo Endoscopic Activity Score, n (%)			
0 (inactive disease)	4 (6.7)		
1 (mild)	23 (38.3)		
2 (moderate)	20 (33.3)		
3 (severe)	13 (21.7)		
Mayo Endoscopic Activity, n (%)			
Remission (0, 1)	27 (45)		
Activation (1, 2)	33 (55)		
Treatments, n (%)			
No treatment	25 (41.7)		
Under treatment	35 (58.3)		
Mayo Clinical Activity Score, n (%)			
Remission (score of ≤2)	13 (21.7)		
Activation (score of >2)	47 (78.3)		
Histology activity index, median(range)	6 (0–8)		

SD: Standard deviation; IBD: inflammatory bowel disease; ESR: erythrocyte sedimentation rate; CRP: C-reactive protein; VEGF: vascular endothelial growth factor;

* Student t-test;

& Mann–Whitney U test;

¥ chi-square test.

**Table 2:** Serum VEGF and calprotectin values according to disease phenotype and treatment status in patients with ulcerative colitis.

		n	%	VEGF	p	Calprotectin	p
Mayo Clinical Activity Score	Remission (≤2)	13	21.7	793 (126–5368)	< 0.001&	340 (101–2648)	0.001&
	Activation (>2)	33	55	2680 (273–5783)		1100 (100–2524)	
Family history of IBDs	Absent	10	16.7	2139 (126–5783)	0.781&	924 (99–2648)	0.746*
	Present	50	83.3	2580 (720–4980)		1143 (101–2520)	
Extraintestinal manifestations	Absent	54	50	2221 (126–5783)	0.573*	963 (99–2648)	0.613*
	Present	6	10.0	1833 (572–5499)		861 (200–1998)	
Treatment status	No treatment	25	41.7	1914 (701–5783)	0.840&	927 (101–2524)	0.551*
	Under treatment	35	58.3	2344 (126–5499)		937 (99–2648)	
Extent of ulcerative colitis	Limited disease	41	68.3	1867 (126–5368)	0.001&	801 (99–2648)	0.012&
	Extensive olitis	19	31.7	2875 (840–5783)		1313 (150–2524)	

VEGF: Vascular endothelial growth factor; IBDs: inflammatory bowel diseases;

* Student t-test;

& Mann–Whitney U test.

**Table 3:** Correlations between serum VEGF and calprotectin values and the clinical and laboratory findings of patients with ulcerative colitis.

Ulcerative colitis patients (n = 60)	VEGF (ng/L)		Calprotectin (ng/L)	
	rho/r	p	rho/r	p
Calprotectin (ng/L)	0.819&	<0.001		
MCS	0.645&	<0.001	0.502&	<0.001
HAI	0.649&	<0.001	0.515&	<0.001
Disease duration (years)	−0.079&	0.549	−0.141&	0.283
MES	0.588&	<0.001	0.489&	<0.001
CRP (mg/L)	0.650&	<0.001	0.468&	<0.001
ESR (mm/h)	0.543&	<0.001	0.426&	0.001
Leukocytes (×10^3^/μL)	0.350&	0.006	0.139&	0.289
Neutrophils (×10^3^/μL)	0.367&	0.004	0.183&	0.161
Platelets (×10^3^/mm3)	0.497&	<0.001	0.431*	0.001

VEGF: Vascular endothelial growth factor; MCS: Mayo Clinical Score; HAI: histology activity index; MES: Mayo Endoscopic Score; CRP: C-reactive protein; ESR: erythrocyte sedimentation rate;

& Spearman correlation coefficient (rho);

* Pearson correlation coefficient (r).
